# Patients with Hepatitis C Infection and Normal Liver Function: A Neuropsychological and Neurophysiological Assessment of Cognitive Functions

**DOI:** 10.1155/2021/8823676

**Published:** 2021-05-19

**Authors:** Jefferson Abrantes, Daniel Simplicio Torres, Carlos Eduardo Brandão-Mello

**Affiliations:** ^1^Department of Neurology, Federal University of the State of Rio de Janeiro, Rua Mariz e Barros, 775-Tijuca, Rio de Janeiro 20270-004, Brazil; ^2^Department of Gastroenterology, Federal University of the State of Rio de Janeiro, 20270-004, Brazil

## Abstract

Several studies have proposed a link between chronic hepatitis C virus (HCV) infection and the development of cognitive disorders. However, the inclusion of confounding factors in their samples significantly limits the interpretation of the results. Therefore, here, we aimed to compare the neurophysiological and cognitive performance between patients with HCV infection and a control group after excluding other factors that may cause cognitive impairment. This cross-sectional, group-control, observational study was performed from September 12, 2014, to October 20, 2017. HCV-infected patients and healthy individuals between 18 and 77 years were considered eligible. The exclusion criteria included well-established causes of cognitive impairment, such as depression and cirrhosis. The participants were submitted to neuropsychological testing to evaluate global cognitive function (minimental), sustained attention, divided attention, selective attention, working memory, psychomotor speed, and executive function and to a neurophysiological evaluation using quantitative electroencephalograms and P300 cognitive evoked potentials. Among the 309 patients considered eligible for the study, we excluded 259 patients who had one or more characteristics from the preestablished exclusion criteria, 18 who did not undergo neuropsychological and neurophysiological testing, and five who exhibited depression. The final sample consisted of 27 patients each in the HCV and control groups. The groups did not differ in age, schooling, and sex. The patients in the HCV group exhibited poorer performances in the cognitive areas involving attention (*p* = 0.01), memory (*p* = 0.02), and psychomotor velocity (*p* = 0.04) apart from exhibiting prolonged latency in the P3b component (*p* = 0.03) and *Z* score (*p* = 0.02) of the P300 evoked cognitive potential. In this study performed with strict selection criteria, on conducting neuropsychological and neurophysiological evaluations, we detected the presence of cognitive impairment characterized by the involvement of attention, working memory, psychomotor processing speed, and memory in the HCV group.

## 1. Introduction

The occurrence of cognitive alterations in patients with hepatopathies has been extensively documented in cases of hepatic encephalopathy and minimal hepatic encephalopathy. However, with the emergence of hepatitis C virus (HCV) infection, a growing body of evidence has demonstrated that cognitive changes may also occur before the development of liver cirrhosis [1, 2].

Patients infected with HCV often report neuropsychiatric complaints even before the development of significant liver changes. Among the frequently reported neuropsychiatric complaints are those regarding the presence of mild cognitive impairment, depression, and fatigue [3]. The prevalence of depression is estimated to be between 20% and 50% in patients infected with HCV, which is higher than the prevalence of approximately 10% that is found in the general population. Another complaint with a prevalence that far exceeds that observed in the general population is fatigue that affects an estimated 50-80% of patients infected with HCV. When we consider the studies published in the literature, we find that up to 50% of the patients present with alterations in the results of neuropsychological tests, and the most significant effects are observed in verbal episodic memory and working memory [4].

The pattern of cognitive impairment that is most often described in HCV infection involves the cortical regions of the frontal lobe and basal ganglia and is a pattern similar to that observed in human immunodeficiency virus (HIV) infection [5]. However, despite the evidence of the presence of cognitive dysfunction in chronic HCV patients, many studies on the subject have included patients with comorbidities, such as cirrhosis and depression, which are also associated with cognitive impairment [6].

Therefore, the objective of the current study was to compare the neurophysiological and cognitive performance between HCV patients and a control group of healthy individuals after excluding any participants with conditions that might interfere with cognitive performance.

## 2. Materials and Methods

### 2.1. Study Design

The current study was an observational, cross-sectional study with a control group.

This study was conducted at the Gastroenterology Service Department of the Gaffrée e Guinle University Hospital (HUGG) in Rio de Janeiro, Brazil, from September 2014 through October 2017.

The research ethics committee of the HUGG approved this study that adhered to the guidelines of the Helsinki Declaration. The study participants completed a free and informed consent form after receiving a detailed explanation of the study.

### 2.2. Inclusion Criteria

All the HCV-infected patients (with detectable serum HCV ribose nucleic acid (RNA)) between the ages of 18 and 77 years who were being treated in the hepatology outpatient clinic were considered eligible. The control group was composed of individuals between the ages of 18 and 77 years who were accompanying the outpatients and inpatients during their visits to the HUGG.

### 2.3. Exclusion Criteria

There was a significant concern associated with the exclusion of conditions that could interfere with the performance in the cognitive test. The exclusion criteria list was extensive, including illiteracy, a history of encephalic vascular accidents, encephalic cranial trauma, dementia, Parkinson's disease, multiple sclerosis, neurodegenerative disease, chronic obstructive pulmonary disease, congestive cardiac insufficiency, other viral infections (HIV, hepatitis B virus (HBV), human T lymphotropic virus (HTLV)), syphilis, significantly compromised liver function, depression, psychiatric illness, illicit drug use, psychotropic drug use, alcoholism, hypothyroidism, cobalamin or folic acid deficiency, and the previous use (in the last 12 months) or current use of interferon.

### 2.4. Neuropsychological Assessment

The cognitive battery was performed in a quiet room with adequate lighting conditions by a neurologist with training in the application of neuropsychological tests who was aware of the serological state of the individuals being tested. All the tests that were applied were translated into Portuguese.

The study participants were asked about the use of illicit drugs, alcoholic beverages, and psychoactive substances 48 h before the neuropsychological testing.

The patients underwent neuropsychological testing, answered questions regarding predefined cognitive complaints (poor memory, dispersal/distractibility, difficulty in performing two tasks simultaneously, and difficulty in driving), and were given additional space to record spontaneous complaints.  Table 1 presents the cognitive tests used and the contemplated cognitive domains.

### 2.5. Depression Screening

A neurologist excluded individuals when a diagnosis of depression was established. In addition to the clinical evaluation, all the study participants were assessed by the Beck-II Depression Inventory (BDI-II).

The BDI-II is one of the most commonly used instruments for measuring the severity of depression and is used in many clinical studies involving patients with chronic diseases, including patients with HCV infection. A score higher than 16 is indicative of significant depressive symptoms [16, 17].

### 2.6. Assessment of Hepatic Involvement

The presence of advanced fibrosis or cirrhosis in a liver biopsy sample (F3 (numerous septa without cirrhosis) or F4 (cirrhosis) in the METAVIR classification system, respectively), transient hepatic elastography (FibroScan), or an aspartate transaminase (AST) to platelet ratio index (APRI) of >1 indicated a significantly compromised liver. Biopsies performed in the 12 months before the study were considered valid for the evaluation of the degree of hepatic impairment.

The APRI represents the relationship between the platelet count and AST level. Levels ≥ 1.5 indicate the presence of significant fibrosis, whereas levels ≤ 0.5 point to the absence of significant fibrosis. Using a cutoff level of ≤1.0, we can rule out the presence of cirrhosis with a sensitivity of 89% and specificity of 75% [18].

### 2.7. Blood Tests

Blood tests were performed on all the participants after the cognitive analysis. The results of serum sodium, thyroid stimulating hormone (TSH), free thyroxine (T4), cobalamin, folic acid, VDRL (Venereal Disease Research Laboratory, syphilis), HIV, HBV, and HTLVI/II tests were analyzed.

### 2.8. Quantitative Electroencephalogram

The equipment used for neurophysiological testing was the BNT-Plus electroencephalogram. To acquire the electroencephalographic record, we used the average adult MEDCAP cap (58-54 cm) that consists of 19 predefined tin electrodes based on the international 10-20 system associated with a ground electrode (Fpz).

The electroencephalogram was recorded with the patients in vigil and with closed eyes while correcting the occurrence of artifacts and ensuring that they avoided drowsiness. The registration time was 20 min. The impedances were kept below 5 kOhms. A 60 Hz notch filter was used, as well as a low-frequency filter of 0.3 Hz and a high frequency filter of 70 Hz.

The electroencephalogram plot was analyzed in order to exclude the presence of artifacts by selecting approximately 20 periods of 2.56 s for a spectral analysis. The data were obtained after the temporal (T)4, parietal (P)4, and occipital (O)2 channel quantification. The following parameters were evaluated: the dominant frequency and average dominant frequency.

In addition to the parameters described above, we analyzed the theta/beta ratio in Cz (central zero) and alpha/theta ratio in O2.

### 2.9. Cognitive Evoked Potentials (P300)

To perform the P300 examinations, we used the 300P sound stimulator that is coupled to the BNT-Plus electroencephalogram apparatus and the Philips SHP2500 headphones to listen to the stimuli generated. We used the average adult MEDCAP cap (58-54 cm) for the recording of the P300 using the following electrodes in the P300 capture assembly: Fpz (prefrontal zero) (ground), Fp1, Fp2, Fz (frontal zero), Cz, and Pz.

The test was performed using the oddball paradigm four times in a row. The oddball paradigm consists of a series of auditory stimuli divided into frequent and rare stimuli [10]. The analyzed data were composed of the latencies of the components, P3a and P3b, as well as the register of the amplitude of the component P3b (P300).

Latency was further analyzed using the age-adjusted *Z* score according to the following formula: *Z* = [value found − (250 + 1.4 × age)]/40. This formula provides a value corresponding to the values suggested by the International Federation of Clinical Neuropsychology [19, 20].

### 2.10. Data Analysis

Since the variables did not exhibit the normal distribution (Gaussian), nonparametric methods were applied based on the rejection of the normality hypothesis by performing the Shapiro-Wilk test in at least one of the groups. The significance criterion adopted was at the level of 5%. The statistical analysis was performed using the software program, *R*, version 3.3.1 (https://www.r-project.org/).

The results of the descriptive analysis were presented in the form of tables; further, regarding the observed data, numerical data were expressed as median, mean, and standard deviation, and categorical data were expressed as frequency (*n*) and percentage (%). The inferential analysis consisted of comparing the variables in the study between the groups of patients and controls and was evaluated by the Mann–Whitney test (nonparametric) in the case of numerical data and by the chi-square test or Fisher's exact test in the case of categorical data.

## 3. Results

### 3.1. Study Sample

#### 3.1.1. Selection of Patients Infected with HCV

A total of 309 medical records were analyzed. This first scrutiny resulted in the exclusion of 259 patients because they presented with one or more characteristics from the previously defined exclusion criteria, of which 224 patients with cirrhosis, 15 patients with HIV, 12 patients using psychotropic drugs, 3 patients with HTLV, 2 patients with epilepsy, 1 patient with sickle cell anemia, 1 patient with HBV, and 1 patient with COPD.

Consultations were scheduled for the remaining 50 patients with the goal of performing neuropsychological and neurophysiological testing. Among these, 18 were absent, and five were excluded due to depression. The remaining 27 patients underwent neuropsychological testing.

#### 3.1.2. Control Group Selection

After evaluating 53 healthy subjects, four participants were excluded because they were using psychotropic drugs, and nine were excluded because they had BDI-II scores of greater than 16 and clinical symptoms of depression. Another thirteen individuals were excluded because they had higher than average levels of schooling compared to those in the HCV group.

#### 3.1.3. Demographic Data

There were no statistically significant differences between the groups in terms of age, schooling, and sex (Table 2).

The comparison of the cognitive complaints between the HCV group and control group did not reveal the presence of any statistically significant differences. However, when asked about the presence of fatigue, 26% (*n* = 7) of the HCV patients affirmed that they experienced this symptom while none of the patients in the control group reported any such symptom (Table 3).

Genotype 1 was responsible for 85.19% of the cases in the HCV group, followed by genotype 2 accounting for 11.11% and genotype 3 that was associated with 3.70%. A total of 11 patients from the HCV group (37.9%) had a history of having received interferon treatment without obtaining a sustained viral response.

A total of 19 patients (70.3%) from the HCV group underwent liver biopsies; subsequently, 16 of them were assigned a METAVIR classification score of F1 (Portal fibrosis without septa), and three were assigned a METAVIR classification score of F2 (portal fibrosis with few septa). All the patients in the HCV group underwent a transient hepatic elastography evaluation (FibroScan); among the total patients, approximately 13 had a METAVIR classification of score F1, and 14 had a METAVIR classification score of F2.

The APRI of the patients in the HCV group was calculated independently of the liver biopsy and fibroscan analysis resulting in a median APRI (± SD) of 0.43 (±0.12). The median (± SD) level of AST in the HCV group was 44.0 (±12.18).

There were no statistically significant differences in the BDI-II scores between the groups. The Mann–Whitney test revealed a median (± SD) score of 9.0 (±5.11) in the HCV group and a median (± SD) score of 8.0 (±4.45) in the control group with *p* = 0.99.

### 3.2. Neuropsychological Comparison between the HCV and Control Groups

**Figure 1:**
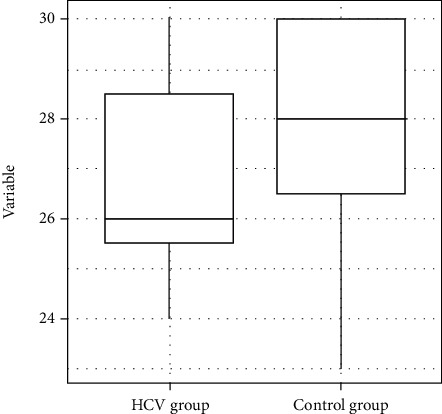
Distribution of the variable Mini-Mental State Examination total scores.

The median (SD) score on the Mini-Mental State Examination was 28.0 (2.03) in the HCV group and 26.0 (2.04) in the control group. After the application of the Mann–Whitney test, it was found that the HCV group exhibited a statistically poorer performance than the control group (*p* = 0.03) (Figure 1).

**Figure 2:**
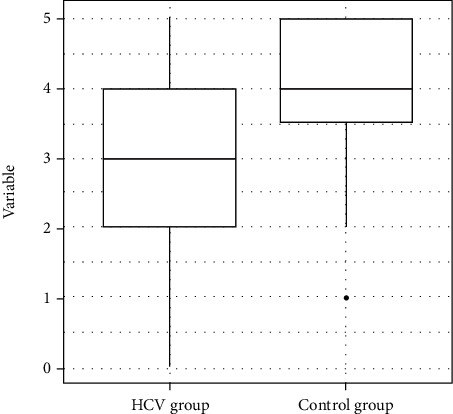
Distribution of the variable Mini-Mental State Examination attention and calculation scores.

**Figure 3:**
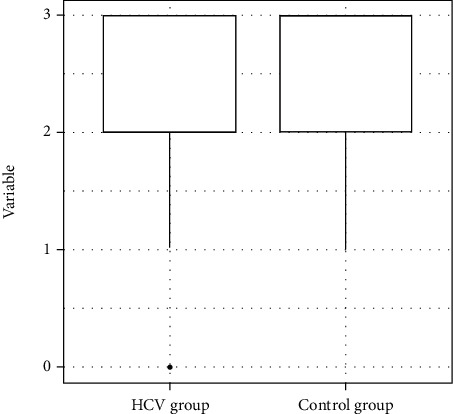
Distribution of the variable Mini-Mental State Examination evocation of word scores.

The statistical analysis revealed that the HCV group exhibited a poorer performance in the items of the Mini-Mental State Examination related to attention (*p* = 0.01), calculation (*p* = 0.01), and word evocation (*p* = 0.02) (Table 4) (Figures 2 and 3).

**Figure 4:**
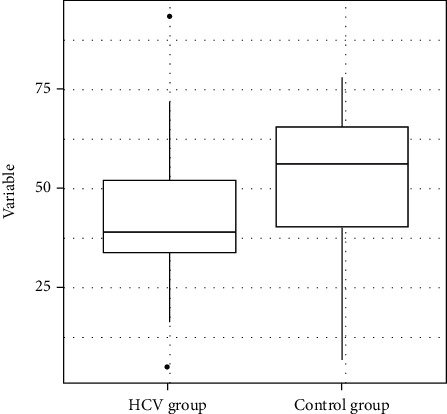
Code testing.

The statistical analysis revealed that the HCV group exhibited a poorer performance in the code test when compared to the control group with a median score (± SD) of 56.0 (±18.81) in the control group and a median score (± SD) of 39 (±17.59) in the HCV group (*p* value = 0.04) (Table 5 and Figure 4).

There were no statistically significant differences between the groups in their performance on the Tower of London test, part A of the Trail Making Test, Part B of the Trail Making Test, Trail Making Test B/A Ratio Score, random letter test, part 1 of the Stroop test, part 2 of the Stroop test, digit test in direct order, and digit test in reverse order (Table 6).

### 3.3. Neurophysiological Comparison between the HCV and Control Groups

**Figure 5:**
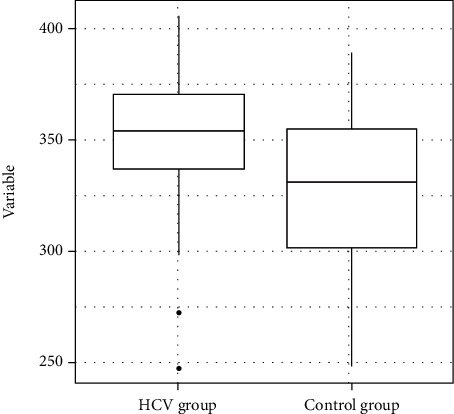
Distribution of variable cognitive evoked potentials (P300) (latency P3b).

**Figure 6:**
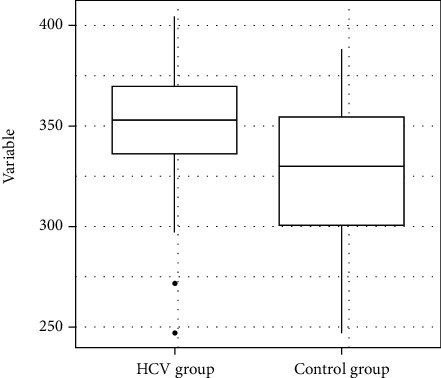
Distribution of variable cognitive evoked potentials (P300) (*Z* score).

The HCV group exhibited prolonged latencies in the P3b component (*p* = 0.03) and *Z* score (*p* = 0.02) when compared to the control group (Table 7, Figures 5 and 6).

There were no statistically significant differences between the HCV and control groups in terms of the theta/beta and alpha/theta frequencies, dominant frequency, and average mean frequency.

No statistically significant differences were observed on evaluating of the HCV group by performed neuropsychological and neurophysiological tests when considering the BDI-II scores and previous use of interferon.

## 4. Discussion

The literature presents evidence of the association between HCV infection and cognitive impairment before developing liver abnormalities. However, several studies have included patients with liver cirrhosis, advanced stage of hepatic fibrosis, and depression; besides, some published papers did not report such associations. Therefore, the controversy about whether HCV infection could be a cause of cognitive alterations remains unknown [6, 21–25].

The inclusion of patients with cirrhosis may introduce undesirable bias into studies on this subject since minimal hepatic encephalopathy patients may present with cognitive changes similar to those reported in patients with HCV infection [26]. Another potential source of selection bias in the cognitive assessment of chronic HCV-infected patients is the inclusion of depressed patients. In this study, there was a great concern to remove such conditions.

In the present study, 70.3% (*n* = 19) of the HCV group underwent liver biopsies, and all the patients underwent transient hepatic elastography and an APRI evaluation, thus minimizing the possibility of the inclusion of patients with advanced liver fibrosis or cirrhosis. The cases involving depression were excluded from the current work based on the clinical criteria assessed by an experienced neurologist. The application of the BDI-II in the HCV group resulted in a median score of 9.00 (±5.11) below the cutoff point of 16 that indicates significant depressive symptoms [7, 8].

Statistical differences were not found between the study sample and control groups in terms of age, schooling, and sex, and the statistical analysis did not reveal a relationship between prior interferon use, as well as the total BDI-II score, with the poorer performance on the psychometric and neurophysiological tests, thus dismissing the possibility that these variables influence the result obtained.

In the neuropsychological evaluation, we observed a worse performance of the HCV group when compared to the control group in the evaluation of the miniexamination of mental status, both in its total score (*p* = 0.03) and in the items attention/calculation (*p* = 0.01) and evocation of words (*p* = 0.02). In the other tests employed, there was a change only in the code test, with lower performance in the HCV group when compared to the control group (*p* = 0.04).

Neurophysiological assessment is a tool that does not suffer direct interference from the sociocultural level as occurs in neuropsychological testing, and its inclusion in this study allows us to assess the impact of these biological markers on this specific population since most of the work involves patients with diseases neurodegenerative disorders such as Alzheimer's or in the evaluation of patients with liver cirrhosis.

The quantitative analysis of the electroencephalogram did not show any statistically significant change between the groups analyzed in the theta/beta relationships in Cz and alpha/theta in O2, as well as there were no changes in the dominant frequency in the different frequency bands analyzed and in the dominant average frequency. Contrary to our expectations, we were going to find an increase in the theta/beta index, as reported in patients with altered attention/concentration.

In this study, the quantitative electroencephalogram did not prove to be a tool capable of detecting changes in the HCV sample without cirrhosis, as demonstrated in studies in patients with liver cirrhosis and minimal hepatic encephalopathy [20, 26] .

In assessing the P300 cognitive evoked potential, we observed an increase in P3b (P300) latency in the HCV group, even after adjusting for the age provided by the *Z* score. There were no differences in amplitude of the P300 and the latency of the P3a component between the groups analyzed.

The neurophysiological and the neuropsychological testing indicated the presence of cognitive impairment involving verbal memory, attention, working memory, and psychomotor speed in the HCV group. When we consider the anatomic-physiological substrate involved in these altered cognitive functions, we can propose that the affected regions are represented by the frontal cortex and posterior regions of the cerebral cortex, with an emphasis on the occipital and parietal lobes.

Our group published a previous study in which no cognitive alterations were detected on comparing HCV carriers to a control group [25]. In this work, we excluded patients with BDI-II scores of above 11, and only 17.2% of the sample had complaints about fatigue.

In the current study, 26% of the patients complained about fatigue, and the median BDI-II score in the present study was 9.0 (±5.11). Therefore, our hypothesis is that the symptoms of depression and fatigue are prodromal manifestations that precede or occur concurrently with a cognitive deficit, and the strict exclusion criteria applied in our previous study probably resulted in an asymptomatic sample that was not representative of the HCV population with cognitive symptoms.

This study has the following limitations: (1) it was a cross-sectional study, which would not be an adequate design to establish a relationship of HCV exposure as a causative factor of cognitive deficit, although the literature available so far is composed of cross-sectional studies [6]; (2) the number of study participants was small, thus limiting the study's statistical power. However, this study's sample size is close to that found in the vast majority of studies available in the literature [6]; and (3) the examiners were not blinded to the participants' serological status, wich may influence the outcome of neuropsychological testing.

## 5. Conclusions

In this study, we observed the presence of cognitive impairment in patients with HCV infection. The observed impairment involved the cognitive areas associated with attention, verbal memory, working memory, and psychomotor processing speed.

Cohort studies comparing patients with HCV infection without hepatic dysfunction and the presence and absence of depression and fatigue may aid in clarifying the hypothesis that the symptoms of depression and fatigue are prodromal manifestations that precede or occur concurrently with a cognitive deficit.

## Figures and Tables

**Table 1:** Cognitive functions and corresponding tests used.

Cognitive function	Tests
Global cognitive function	Mini-Mental State Examination [7, 8]
Working memory	Direct numerical order test (WAIS-III) [9]
Verbal attention	Indirect numerical order test (WAIS-III) [9]
Sustained attention	“A” random letters test [7, 10]
Divided attention	Trail Making Test (parts A and B) [9, 11]
Selective attention	Stroop test (parts 1 and 2) [12, 13]
Nonverbal executive function	London Tower [14]
	Trail Making Test B/A Ratio Score [15]
Psychomotor speed	Code testing (WAIS-III) [9]

**Table 2:** Descriptive statistics for age, education level, and sex.

	Groups		
	Control (*n* = 27)	HCV (*n* = 27)	*p* value
Age (years)			
Mean (± SD)	58 (±10.48)	58 (±12.21)	Mann–Whitney test 0.83
Educational level (years)			
Mean (± SD)	9.7 (±3.11)	11.0 (±3.97)	Mann–Whitney test 0.67
Sex	*n* (%)	*n* (%)	Chi-square test
Male	11 (41%)	12 (44%)	0.99
Female	16 (59%)	15 (56%)	

HCV: hepatitis C virus; SD: standard deviation.

**Table 3:** Cognitive complaints of patients in the HCV and control groups.

Complaints	Group				
	Control (*n* = 27)		HCV (*n* = 27)		*p* value
	*N*	%	*n*	%	Chi-square test
Poor memory	14	52	13	48	0.99
Dispersal/distractibility	12	44	10	37	0.59
Reasoning speed reduction	9	33	8	30	0.45

Abbreviations: HCV: hepatitis C virus.

**Table 4:** Scores of patients with HCV infection and the control group on the items of the Mini-Mental State Examination.

	Control (*n* = 27)		HCV (*n* = 27)		
	Median	Standard deviation	Median	Standard deviation	*p* value
Orientation to time	5.00	0.27	5.00	—	0.16^∗^
Orientation to place	5.00	0.19	5.00	0.46	0.30^∗∗^
Immediate memory	3.00	—	3.00	—	—
Attention and calculation	4.00	1.31	3.00	1.56	0.01^∗^
Word evocation	3.00	0.55	2.00	0.80	0.02^∗^
Nomination	2.00	0.19	2.00	—	0.34^∗∗^
Repetition	1.00	0.19	1.00	0.48	0.10^∗^
Complex commands	3.00	0.42	3.00	0.27	0.98^∗^
Reading	1.00	—	1.00	—	—
Writing	1.00	0.19	1.00	0.27	0.18^∗∗^
Drawing copy	1.00	0.48	1.00	NA	0.13^∗∗^
MMSE-score	28.00	2.03	26.00	2.04	0.03^∗^

^∗^Mann–Whitney *U* test, ^∗∗^Fisher's exact test. Abbreviations: HCV: hepatitis C virus; MMSE: Mini-Mental State Examination.

**Table 5:** Descriptive statistics of code testing.

	Control (*n* = 27)		HCV (*n* = 27)		
	Median	Standard deviation	Median	Standard deviation	*p* value
Code test-correct answer	56.00	18.81	39.00	17.59	0.04^∗^
Code test-wrong	—	0.64	—	0.73	—

^∗^Teste de Mann–Whitney.

**Table 6:** Descriptive statistics of the Tower of London test, trail test, random letter test, Stroop test and digit test (forward and reverse order).

	Control (*n* = 27)		HCV (*n* = 27)		
	Median	Standard deviation	Median	Standard deviation	*p* value
London tower	29.00	5.68	27.00	6.34	0.12^∗^
Trail making part A	47.10	31.96	52.77	19.30	0.10^∗^
Trail making part B	121.00	93.01	149.00	97.54	0.19^∗^
Trail Making B/A Ratio Score	2.36	1.32	2.83	1.43	0.63^∗^
“A” random letters test	—	0.84	—	0.83	0.51^∗^
Stroop test-part 1	101.00	27.10	113.00	43.85	0.14^∗^
Stroop test-part 2	230.00	48.04	300.00	54.87	0.09^∗^
Direct numerical order	8.00	2.89	7.00	2.89	0.20^∗^
Indirect numerical order	4.00	2.76	3.00	NA	0.08^∗^

^∗^Teste de Mann–Whitney.

**Table 7:** Descriptive statistics of the cognitive evoked potential (P300).

Variable	Control (*n* = 27)		HCV (*n* = 27)		
	Median	Standard deviation	Median	Standard deviation	*p* value^∗^
P300 P3a latency	304.00	33.07	330.70	36.59	0.07
P300 P3b latency	330.70	34.38	354.10	37.02	0.03
P300 *Z* score	330.00	34.37	353.30	37.00	0.02
P300 amplitude	8.50	5.43	8.16	4.24	0.20

^∗^Mann–Whitney U test. Abbreviations: HCV: hepatitis C virus.

## Data Availability

There is no complementary data to the article.
